# An In-Hospital Mortality Risk Model for Patients Undergoing Coronary Artery Bypass Grafting Based on Machine Learning: Cohort Study

**DOI:** 10.2196/80671

**Published:** 2026-05-25

**Authors:** Kun Zhu, Wenyuan Lu, Shui Liu, Hongyuan Lin, Jianfeng Hou

**Affiliations:** 1 Cardiac Surgery Centre Fuwai Hospital Chinese Academy of Medical Sciences and Peking Union Medical College Beijing China; 2 Department of Radiology Aerospace Center Hospital Beijing China

**Keywords:** coronary artery bypass grafting, CABG, mortality risk, prediction model, machine learning

## Abstract

**Background:**

Ischemic heart disease remains the leading cause of death worldwide. Coronary artery bypass grafting (CABG) remains the primary surgical treatment for ischemic heart disease. There is currently a lack of highly accurate and widely applicable models for assessing the risk of postoperative mortality following CABG.

**Objective:**

This study aimed to develop and validate an in-hospital mortality risk prediction system for patients undergoing coronary artery bypass grafting (CABG) by using machine learning algorithms and to compare its performance with the European System for Cardiac Operative Risk Evaluation II (EuroSCORE II) and Sino System for Coronary Operative Risk Evaluation (SinoSCORE).

**Methods:**

Between January 2017 and December 2020, 21,443 patients undergoing CABG in the Chinese Cardiac Surgery Registry were included. Patients were randomly divided into training (n=17,753) and test (n=3690) cohorts. We addressed class imbalance using the synthetic minority oversampling technique (SMOTE) and optimized hyperparameters via grid search. Fifteen machine learning algorithms were developed to predict in-hospital mortality. Performance was evaluated using the area under the receiver operating characteristic curve (AUC), calibration metrics (Brier score), and decision curve analysis, and was compared against EuroSCORE II and SinoSCORE.

**Results:**

A total of 21,443 patients were included. Overall, in-hospital mortality was 2.1% (n=450). The Extreme Gradient Boosting (XGBoost) model achieved the best performance with an AUC of 0.850 in the training cohort and 0.782 in the independent test cohort (this cohort was independent and not involved in model construction). While EuroSCORE II showed an AUC of 0.722 and SinoSCORE showed an AUC of 0.726 in the test cohort, the XGBoost model demonstrated superior discrimination and calibration (*P*<.05).

**Conclusions:**

Our study developed and validated a machine learning–based risk prediction model for in-hospital mortality after CABG by using a large-scale Chinese multicenter registry. Among the algorithms tested, the XGBoost model demonstrated superior discrimination and calibration compared with the traditional EuroSCORE II and SinoSCORE, suggesting that locally calibrated models may better capture the risk profile of Chinese patients. The derived 7-variable web calculator may serve as an exploratory auxiliary tool to provide a preliminary reference for bedside risk stratification, though its direct impact on surgical decision-making requires further prospective validation. Future research should focus on independent test cohorts across diverse hospital tiers to ensure broad generalizability.

## Introduction

Ischemic heart disease remains the leading cause of death worldwide [1]. In 2021, it was estimated to affect 250 million individuals, resulting in 9.21 million deaths worldwide. With the increase in life expectancy and the aging of the population, the prevalence of ischemic heart disease and the number of deaths increased by 71.55% and 126.67%, respectively, from 1990 to 2021 [2]. Coronary artery bypass grafting (CABG) remains the primary surgical treatment for ischemic heart disease [3,4]. According to the Society of Thoracic Surgeons (STS) Adult Cardiac Surgery Database (ACSD), isolated CABG remains the most commonly performed cardiac surgical procedure, with approximately 290,000 cases per year, while operative mortality has declined to 1.91% [5]. The continued decrease relies on improvements in the quality of care and the use of surgical risk assessment systems.

With the accumulation of CABG surgeries, the establishment of large-scale databases (eg, European System for Cardiac Operative Risk Evaluation [EuroSCORE] [6], STS ACSD [5,7], and Chinese Cardiac Surgery Registry [CCSR] [8]), and the increasing application of statistical methods such as logistic regression, a series of prediction models for operative mortality risk applicable to CABG have been developed. However, these models have limitations, including poor discrimination, accuracy, and applicability, and they generally overestimate operative mortality risk in China [9-12]. There is an urgent need for a new risk assessment system that can address the limitations of traditional methods and be applied to current clinical work.

Machine learning, a fundamental aspect of artificial intelligence (AI), relies on analyzing training data to identify patterns and relationships between input and output variables [13]. As a rapidly advancing technology, its use in medicine is expanding [14,15]. Machine learning aids clinicians by enhancing their understanding of diseases and enabling the development of personalized prevention and treatment plans through the statistical analysis and aggregation of extensive clinical data. Machine learning has proven to be highly effective in assessing the risk of operative mortality and complications in cardiovascular surgery [16-18]. Its performance shows significant promise and broad potential for future applications.

This study aims to investigate in-hospital mortality and risk factors following CABG by retrospectively analyzing clinical data from patients included in the CCSR, constructing a prediction model using various machine learning algorithms and comparing its performance with traditional models such as EuroSCORE Ⅱ, to offer a new perspective on assessing operative mortality after CABG.

## Methods

### Study Population

The CCSR database includes data from consecutive patients undergoing cardiac surgery at 87 participating centers located in nearly all provinces and directly controlled municipalities in China [8].

Patients included in the CCSR were selected from this database between January 2017 and December 2020. Inclusion criteria were patients who underwent CABG surgery with or without other cardiovascular surgeries and were aged 18 years or older. Exclusion criteria were incomplete surgical treatment or incomplete medical records. For grouping, enrolled patients were randomly divided into a training cohort (n=17,753) for model development and an independent test cohort (n=3690) for final performance evaluation. Within the training cohort, a 10-fold cross-validation strategy was used, where the data were iteratively split into 9 folds for training and 1-fold for internal validation to optimize hyperparameters via grid search.

### Data Collection

Candidate risk factors included patient demographic characteristics, medical history and comorbidities, hospital evaluation and workup, and procedure-related factors. Patient demographic characteristics included age, sex, BMI, and tobacco use. Medical history and comorbidities included hypertension, diabetes mellitus, dyslipidemia, cerebrovascular accident, chronic kidney disease (CKD), chronic obstructive pulmonary disease (COPD), peripheral vascular disease (PVD), perioperative atrial fibrillation (AF), angina, prior myocardial infarction (MI), prior heart failure (HF), prior percutaneous coronary intervention (PCI), and prior cardiac surgery. Hospital evaluation and workup included laboratory results such as total cholesterol, low-density lipoprotein, fasting blood glucose, and serum creatinine. The creatinine clearance rate (CCr) was calculated using the Cockcroft-Gault formula. Additional variables included left ventricular ejection fraction (LVEF), left ventricular end-diastolic diameter (LVEDD), left atrial dimension, valve lesions, New York Heart Association (NYHA) classification, Canadian Cardiovascular Society (CCS) angina classification, and critical preoperative state. Critical preoperative state was defined as any one of the following during the same hospital admission: cardiogenic shock, cardiopulmonary resuscitation, ventricular fibrillation or flutter, or intra-aortic balloon pump implantation. Procedure-related factors included urgency of surgery and types of combined procedures.

The primary study end point was in-hospital mortality, defined as all-cause in-hospital death after CABG.

### Statistical Analysis

In our study, statistical analyses were conducted using SPSS (version 26.0; IBM Corp). Machine learning prediction models were developed using Python (version 3.10; Python Software Foundation). Categorical variables were presented as frequencies and compared using the chi-square test or Fisher exact test for groups of unordered categorical variables. The Mann-Whitney *U* test was used for comparisons between groups of ordered categorical variables (ranked variables). Continuous variables were tested for normality using the Kolmogorov-Smirnov method. Normally distributed continuous variables were presented as mean (SD) and compared using the Student *t* test. Nonnormally distributed data were presented as median (IQR) and compared using the Mann-Whitney *U* test. Two-sided tests were used for all statistical analyses, and *P*<.05 was considered statistically significant.

### Machine Learning Models Development

In this study, we built a machine learning model with predictive accuracy through data preprocessing, feature selection, model construction, model validation, and independent testing (this cohort was independent and not involved in model construction).

The model development pipeline was implemented in Python 3.10, comprising data preprocessing, feature selection, and model construction. In the preprocessing phase, continuous variables were standardized using *z* score normalization to ensure feature comparability. Missing values in continuous variables were imputed using the median of the nonmissing values, while patients with incomplete data regarding the primary end point were excluded. To address the substantial class imbalance (in-hospital mortality rate of 450/21,443, 2.1%), we applied the synthetic minority oversampling technique (SMOTE) to the training cohort during the learning phase. This ensured that the algorithms were not biased toward the majority class (survival) and improved the sensitivity for detecting mortality events.

Feature selection was conducted using a rigorous 2-step strategy to identify robust predictors and reduce dimensionality. In the first stage, feature stability was evaluated by calculating the intraclass correlation coefficient (ICC) across 100 random subsamples of the training cohort. This step aimed to filter out variables with poor reproducibility or high susceptibility to noise; only features with an ICC>0.90 were retained as stable candidates. In the second stage, these stable variables were entered into a least absolute shrinkage and selection operator (LASSO) regression model with 10-fold cross-validation. The LASSO algorithm, using L1 regularization, effectively eliminated multicollinearity and identified the final 7 key clinical features with the strongest association to in-hospital mortality. This process effectively filtered out redundant variables and identified key clinical features with the strongest association to the outcome, which were then used for model training.

We developed and compared 15 machine learning algorithms, including logistic regression, random forest (RF), support vector machine, and Extreme Gradient Boosting (XGBoost). Hyperparameters for the tree-based models (specifically max_depth, learning_rate, gamma, and n_estimators for XGBoost) were optimized using a grid search strategy with 10-fold cross-validation on the training cohort. Model performance was quantified using the area under the receiver operating characteristic curve (AUC). To address the reviewer’s recommendation, we also evaluated model calibration using the Brier score and calibration plots (slope and intercept) to assess the agreement between predicted probabilities and observed outcomes in the independent test cohort. Using these methods, we obtained a machine learning model with high accuracy that is able to better predict the mortality risk in patients with different clinical backgrounds. This study provides important methodological guidance for developing real-world applications based on optimal models in clinical practice.

### Ethical Considerations

The CCSR database was generated in 2013, including 87 large cardiac centers across the country with routine data submission, strict data audits, and instant feedback. These sites are the leading cardiac centers in their respective regions and have many features that are common among large cardiac care centers in China. The study was conducted in accordance with the Declaration of Helsinki and was approved by the institutional review board of Fuwai Hospital (protocol code 2021-1477; approved on August 11, 2021). This requirement for informed consent was waived. Identifiable features of research participants or users in any images of the manuscript or supplementary material are not visible.

## Results

### Baseline Information

**Figure 1:**
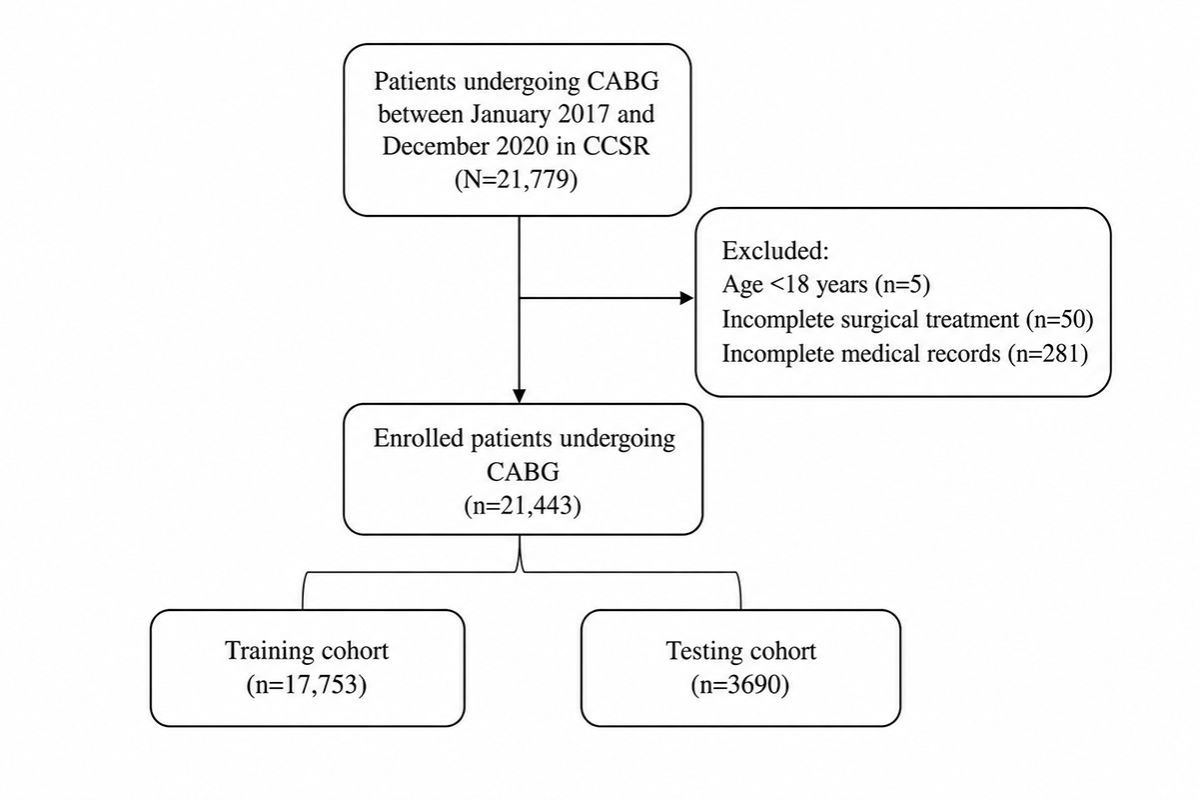
Flowchart of patient enrollment and final sample division. CABG: coronary artery bypass grafting; CCSR: Chinese Cardiac Surgery Registry.

A total of 21,443 patients undergoing CABG between January 2017 and December 2020 met the study criteria and were included in this analysis (Figure 1). Of these, 16,197 (75.5%) were male and 5246 (24.5%) were female patients, with a median age of 63 (IQR 56-68) years. The median BMI was 24.91 (IQR 22.84-27.06) kg/m^2^. Left main disease was present in 6007 (28%) patients and three-vessel disease in 16,583 (77.3%; Table 1).

In-hospital mortality in the overall cohort was 2.1% (450/21,443). Regarding patient factors, patients who died and those who survived differed significantly according to age, tobacco use, dyslipidemia, CKD, COPD, PVD, perioperative AF, prior HF, prior cardiac surgery, NYHA and CCS classification, left main disease, and other variables.

**Table 1:** Patient demographics and clinical features.

Variables			Overall (N=21,443)		Death (n=450)		Survival (n=20,993)		*P* value	
Age (y), median (IQR)			63 (56-68)		66 (61-72)		63 (56-68)		*<.001* ^a^	
Male, n (%)			16,197 (75.5)		354 (78.7)		15,843 (75.5)		.12	
BMI (kg/m^2^), median (IQR)			24.91 (22.84-27.06)		24.22 (22.49-26.41)		24.91 (22.86-27.06)		.78	
Tobacco use, n (%)			11,782 (54.9)		212 (47.1)		11,570 (55.1)		*.003*	
Hypertension, n (%)			13,070 (61)		277 (61.6)		12,793 (60.9)		.94	
Diabetes mellitus, n (%)			6891 (32.1)		153 (34)		6738 (32.1)		.39	
Insulin-dependent diabetes mellitus, n (%)			2621 (12.2)		50 (11.1)		2571 (12.2)		.47	
Dyslipidemia, n (%)			7030 (32.8)		94 (20.9)		6936 (33)		*<.001*	
CKD^b^, n (%)			2889 (13.5)		42 (9.3)		2847 (13.6)		*<.001*	
COPD^c^, n (%)			292 (1.4)		10 (2.2)		282 (1.3)		*.02*	
PVD^d^, n (%)			726 (3.4)		21 (4.7)		705 (3.4)		*.002*	
Cerebrovascular accident, n (%)			2185 (10.2)		71 (15.8)		2114 (10.1)		*<.001*	
HF^e^, n (%)			1020 (4.8)		61 (13.6)		959 (4.6)		*<.001*	
**CCS^f^** **class, n (%)**										*<.001*
	None	1998 (9.3)		65 (14.4)		1933 (9.2)				
	CCS Ⅰ	3448 (16.1)		83 (18.4)		3365 (16)				
	CCS Ⅱ	4725 (22)		90 (20)		4635 (22.1)				
	CCS Ⅲ	8988 (41.9)		125 (27.8)		8863 (42.2)				
	CCS Ⅳ	880 (4.1)		37 (8.2)		843 (4)				
**NYHA^g^** **class, n (%)**										*<.001*
	Ⅰ	965 (4.5)		35 (7.8)		930 (4.4)				
	Ⅱ	2776 (12.9)		93 (20.7)		2683 (12.8)				
	Ⅲ	16,103 (75.1)		249 (55.3)		15,854 (75.5)				
	Ⅳ	1575 (7.3)		72 (16)		1503 (7.2)				
Perioperative AF^h^, n (%)			984 (4.6)		43 (9.6)		941 (4.5)		*<.001*	
Prior MI^i^, n (%)			5423 (25.3)		143 (31.8)		5280 (25.2)		*.006*	
Prior PCI^j^, n (%)			2463 (11.5)		63 (14)		2400 (11.4)		.23	
Prior cardiac surgery, n (%)			703 (3.3)		29 (6.4)		674 (3.2)		*<.001*	
Left main disease, n (%)			6007 (28)		142 (31.6)		5865 (27.9)		*<.001*	
Three-vessel disease, n (%)			16,583 (77.3)		338 (75.1)		16,245 (77.4)		.26	
SCr (μmol/L), median (IQR)			78.00 (67.00-92.00)		85.00 (72.00-106.50)		78.00 (67.00-91.70)		*<.001*	
CCr^k^ (mL/min/1.73 m^2^), median (IQR)			80.40 (64.37-99.07)		67.07 (49.62-84.29)		80.68 (64.70-99.36)		*<.001*	
TC^l^ (mmol/L), median (IQR)			3.82 (3.24-4.60)		3.67 (3.10-4.64)		3.83 (3.24-4.60)		.59	
LDL^m^ (mmol/L), median (IQR)			2.30 (1.80-2.92)		2.27 (1.77-2.98)		2.30 (1.80-2.92)		.34	
FBG^n^ (mmol/L), median (IQR)			5.61 (4.90-7.01)		5.91 (5.00-7.31)		5.60 (4.90-7.00)		*.007*	
LVEF^o^ (%), median (IQR)			58.0 (47.0-63.0)		52.9 (40.0-61.06)		58.0 (48.0-63.0)		*<.001*	
LVEDD^p^ (mm), median (IQR)			49.0 (42.0-55.0)		52.0 (47.9-59.0)		48.3 (41.7-55.0)		*<.001*	
LAD^q^ (mm), median (IQR)			37.0 (32.0-42.0)		40.0 (35.0-45.0)		37.0 (32.0-42.0)		*<.001*	
Critical preoperative state, n (%)			1291 (6)		102 (22.7)		1189 (5.7)		*<.001*	
Aortic stenosis, n (%)			725 (3.4)		30 (6.7)		695 (3.3)		*<.001*	
Aortic insufficiency, n (%)			6047 (28.2)		190 (42.2)		5857 (27.9)		*<.001*	
Mitral stenosis, n (%)			525 (2.4)		20 (4.4)		505 (2.4)		*<.001*	
Mitral insufficiency, n (%)			9659 (45)		293 (65.1)		9366 (44.6)		*<.001*	
Tricuspid stenosis, n (%)			10 (0)		0 (0)		10 (0)		*<.001*	
Tricuspid insufficiency, n (%)			7110 (33.2)		216 (48)		6894 (32.8)		*<.001*	
Nonelective surgery, n (%)			393 (1.8)		79 (17.6)		314 (1.5)		*<.001*	
Combined valve surgery, n (%)			3335 (15.6)		137 (30.4)		3198 (15.2)		*<.001*	
Combined surgery except valve, n (%)			2217 (10.3)		105 (23.3)		2112 (10.1)		*<.001*	

^a^Italicized values denote *P*<.05.

^b^CKD: chronic kidney disease.

^c^COPD: chronic obstructive pulmonary disease.

^d^PVD: peripheral vascular disease.

^e^HF: heart failure.

^f^CCS: Canadian Cardiovascular Society.

^g^NYHA: New York Heart Association.

^h^AF: atrial fibrillation.

^i^MI: myocardial infarction.

^j^PCI: percutaneous coronary intervention.

^k^SCr: serum creatinine.

^l^CCr: creatinine clearance rate.

^m^LDL: low-density lipoprotein.

^n^FBG: fasting blood glucose.

^o^LVEF: left ventricular ejection fraction.

^p^LVEDD: left ventricular end-diastolic diameter.

^q^LAD: left atrial dimension.

### Establishment of the Machine Learning Model

According to the results of feature screening and possible clinical significance, the factors screened for the prediction models were age, gender, BMI, tobacco use, hypertension, diabetes mellitus, dyslipidemia, CKD, COPD, PVD, prior cerebrovascular accident, prior HF, CCS class, NYHA class, perioperative AF, prior MI, prior PCI, prior cardiac surgery, serum creatinine, CCr, total cholesterol, low-density lipoprotein, fasting blood glucose, LVEF, LVEDD, combined cardiac surgery, critical preoperative state, and other variables.

**Figure 2:**
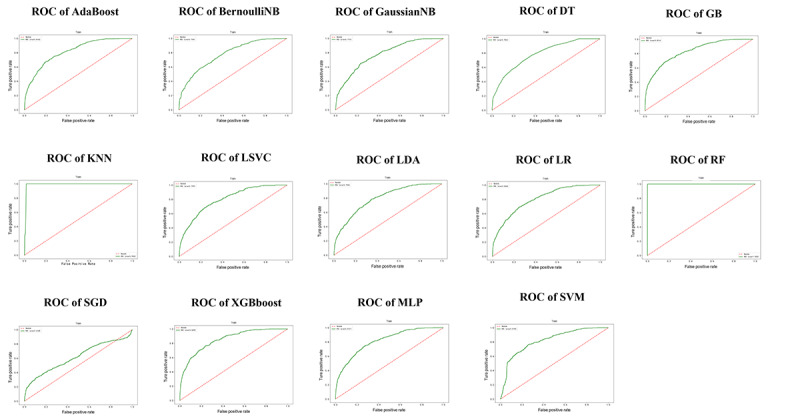
Receiver operating characteristic (ROC) of the machine learning model in the training cohort. The perfect area under the ROC curve (AUC) (1.00) observed for random forest (RF) and k-nearest neighbor (KNN) indicates overfitting on the training data; the model’s true generalizability should be interpreted based on the test cohort results (Figure 7). AdaBoost: adaptive boosting; BernoulliNB: Bernoulli Naive Bayes; DT: decision tree; GB: gradient boosting; GaussianNB: Gaussian Naive Bayes; LDA: linear discriminant analysis; LR: logistic regression; LSVC: linear support vector classification; MLP: multilayer perceptron; SGD: stochastic gradient descent; SVM: support vector machine; XGBoost: Extreme Gradient Boosting.

**Figure 3:**
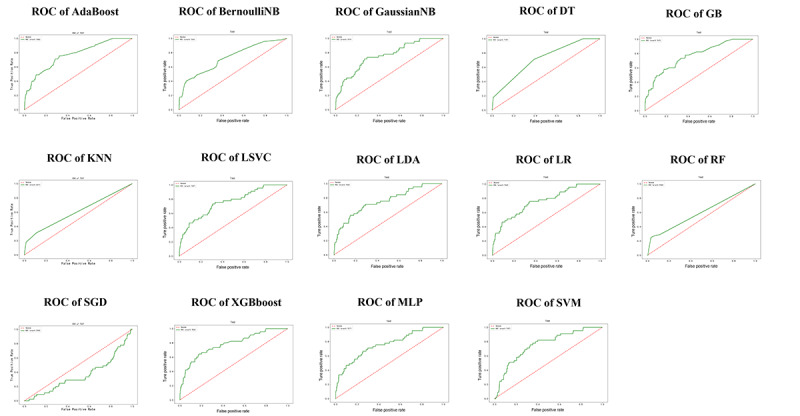
Receiver operating characteristic (ROC) of the machine learning model in the validation cohort. AdaBoost: adaptive boosting; BernoulliNB: Bernoulli Naive Bayes; DT: decision tree; GB: gradient boosting; GaussianNB: Gaussian Naive Bayes; KNN: k-nearest neighbor; LDA: linear discriminant analysis; LR: logistic regression; LSVC: linear support vector classification; MLP: multilayer perceptron; RF: random forest; SGD: stochastic gradient descent; SVM: support vector machine; XGBoost: Extreme Gradient Boosting.

**Figure 4:**
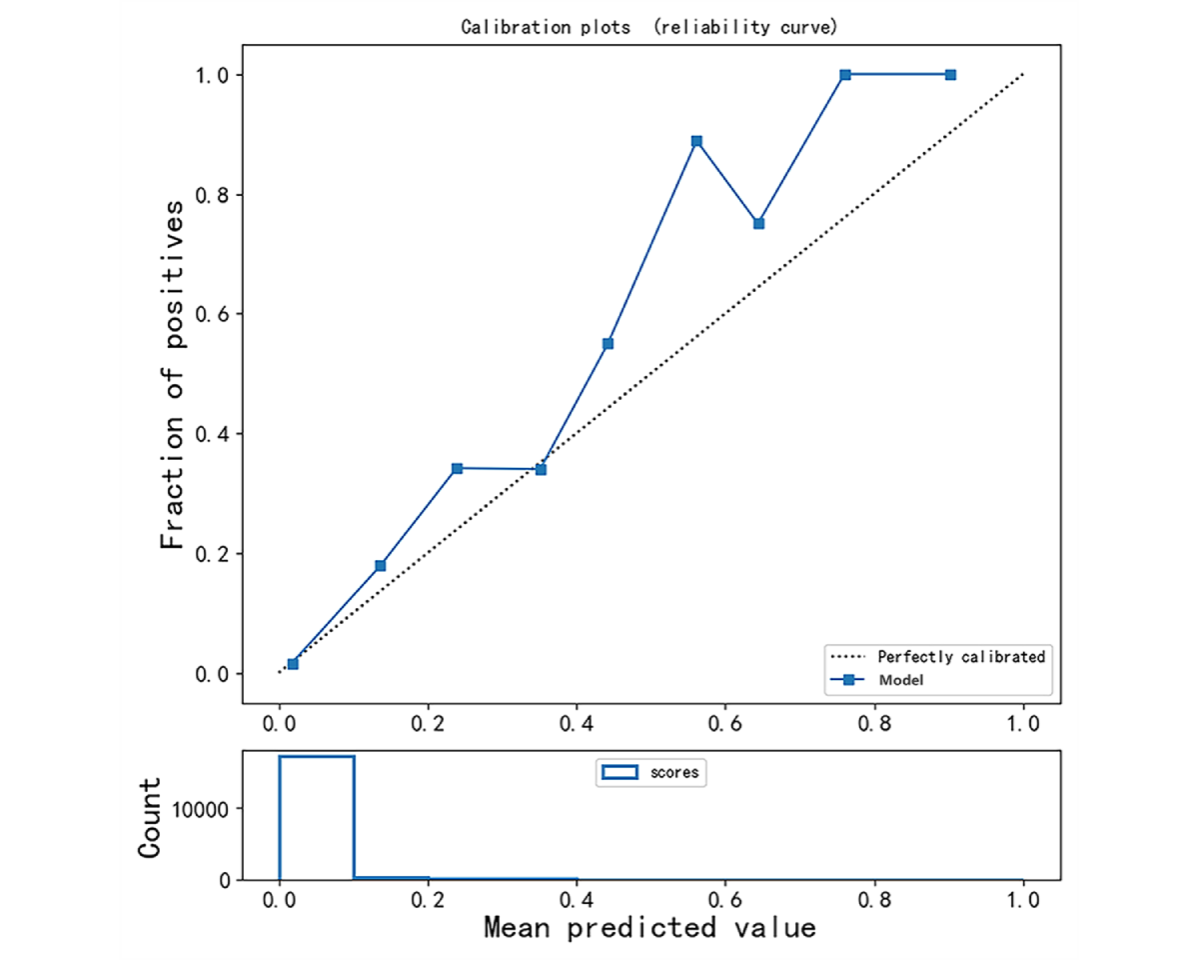
Calibration curve of the Extreme Gradient Boosting (XGBoost) model in the training cohort.

**Figure 5:**
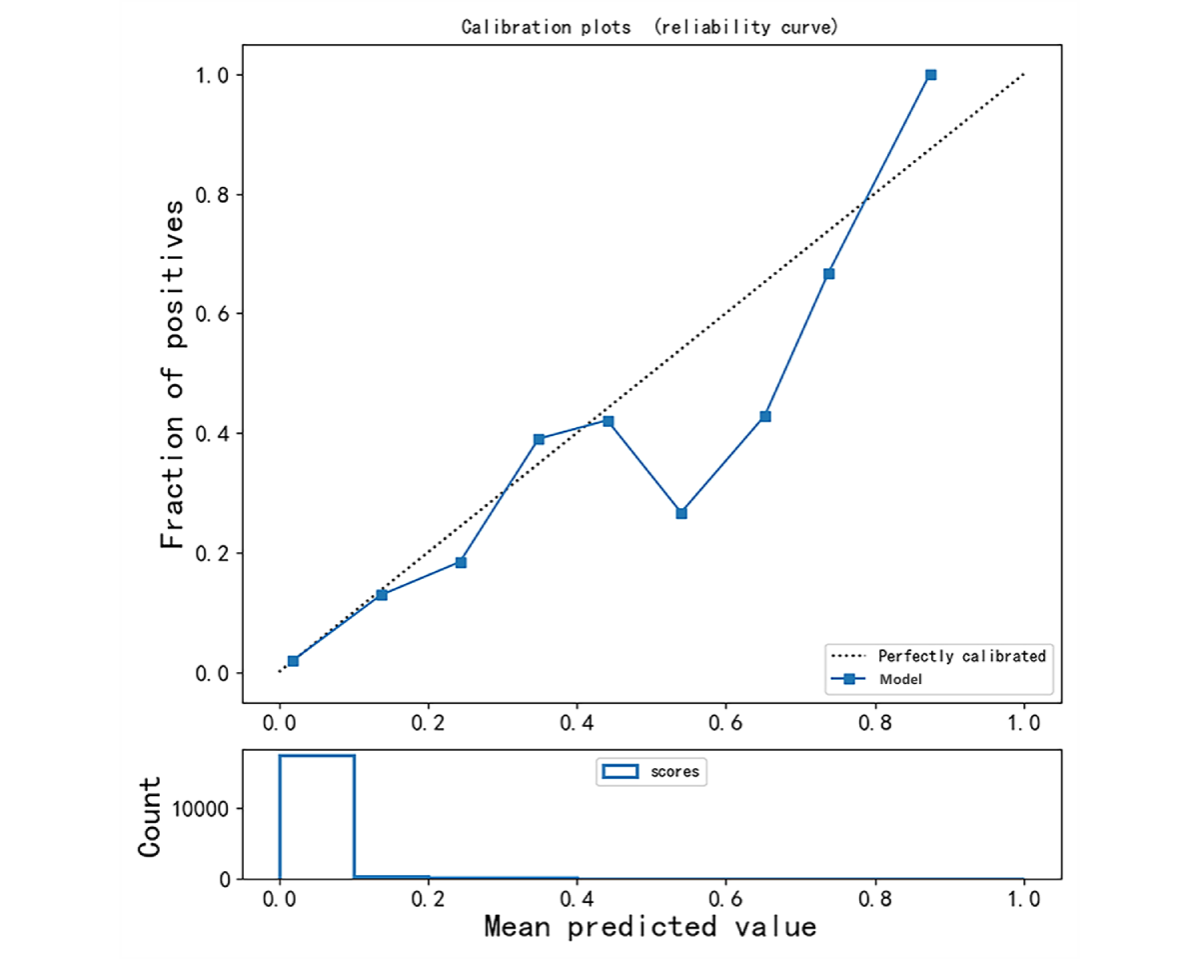
Calibration curve of the Extreme Gradient Boosting (XGBoost) model in the validation cohort.

**Figure 6:**
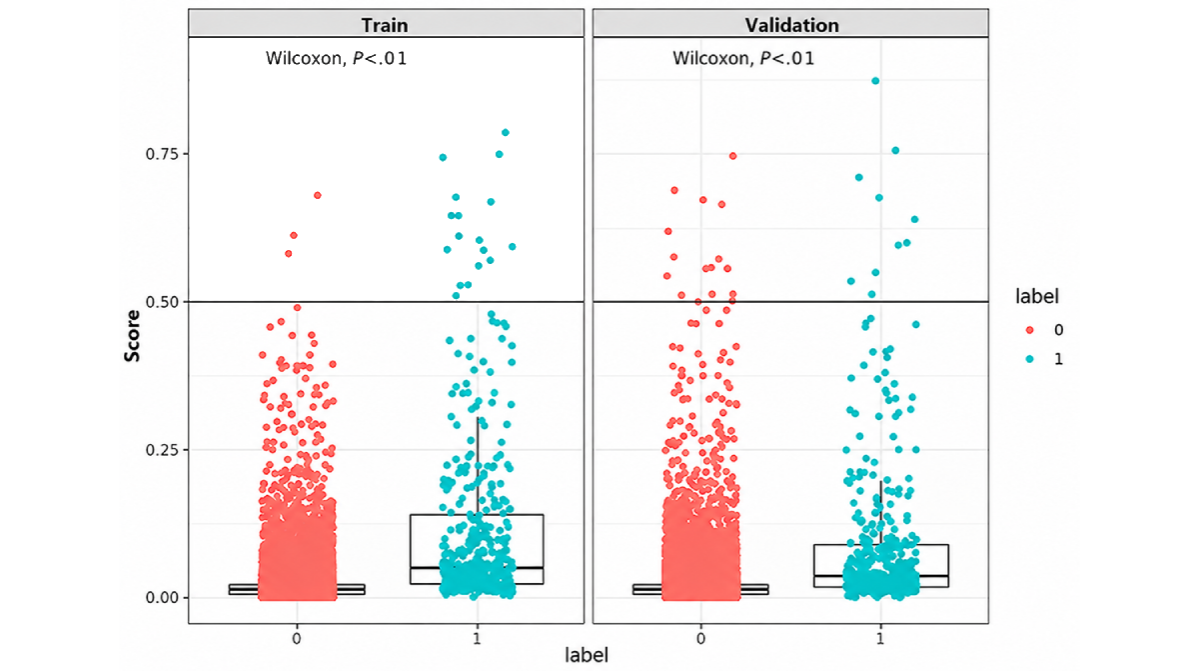
The box plot shows that there are significant differences between the output of the Extreme Gradient Boosting (XGBoost) model predicted to survival and death.

In the training cohort, 10-fold cross-validation was applied for model building and validation. Tables 2 and 3 show the predictive performance of each machine learning model in the training and validation cohorts. The XGBoost model had the best discrimination and calibration. In the training and validation cohorts, the AUC values were 0.850 (95% CI 0.832-0.868) and 0.798 (95% CI 0.777-0.819), respectively (Figures 2 and 3). Performance metrics reported for the validation cohort represent the average diagnostic accuracy across the 10-fold cross-validation within the training data. The XGBoost model calibration curve in the training and validation cohorts is shown in Figures 4 and 5. Variables included in the XGBoost model included prior HF, critical preoperative state, CCr, LVEDD, MI, combined valve surgery, and nonelective surgery. Figure 6 shows that there are significant differences between the output of the XGBoost model predicted for survival and death.

**Figure 7:**
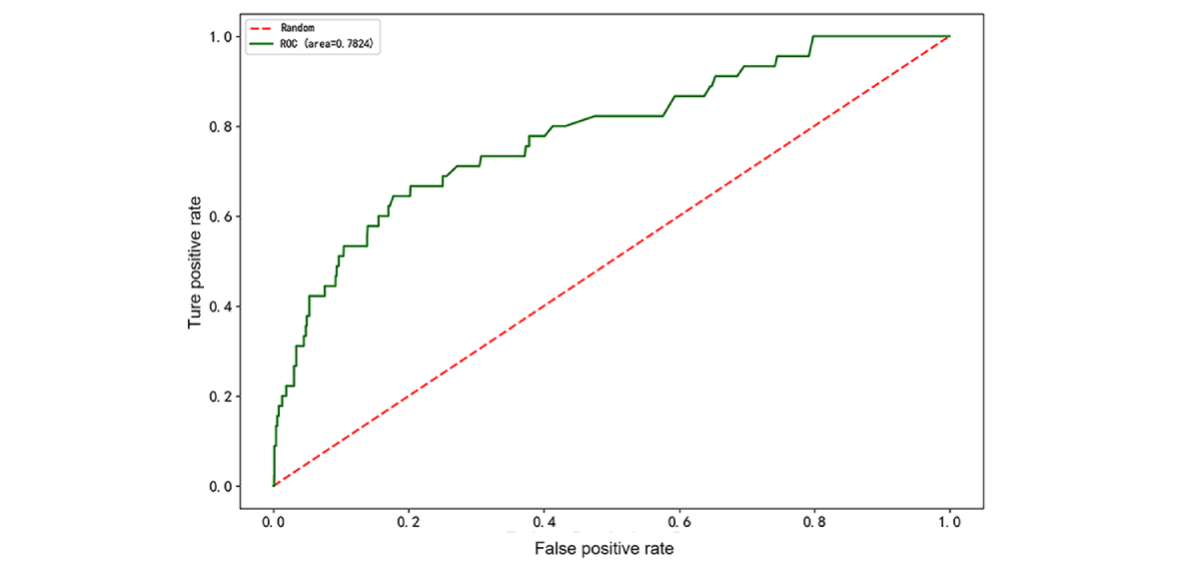
Receiver operating characteristic (ROC) of the Extreme Gradient Boosting (XGBoost) model in the test cohort.

We independently validated the XGBoost prediction model, which showed the best performance in the training and validation cohorts. In the test cohort, it achieved an AUC of 0.782 (95% CI 0.709-0.855; Figure 7), demonstrating its stability and accuracy in real-world applications. Furthermore, the calibration assessment in the test cohort yielded a Brier score of 0.019, a calibration slope of 0.94, and an intercept of −0.05, indicating that the predicted probabilities were well aligned with the observed mortality rates. In comparison, the AUC values of EuroSCORE II were 0.769 (95% CI 0.743-0.793) in the training cohort and 0.722 (95% CI 0.633-0.811) in the test cohort. The AUC values of Sino System for Coronary Operative Risk Evaluation (SinoSCORE) were 0.746 (95% CI 0.718-0.773) in the training cohort and 0.726 (95% CI 0.700-0.753) in the test cohort, indicating that the XGBoost model outperformed EuroSCORE II and SinoSCORE (*P*<.05).

**Figure 8:**
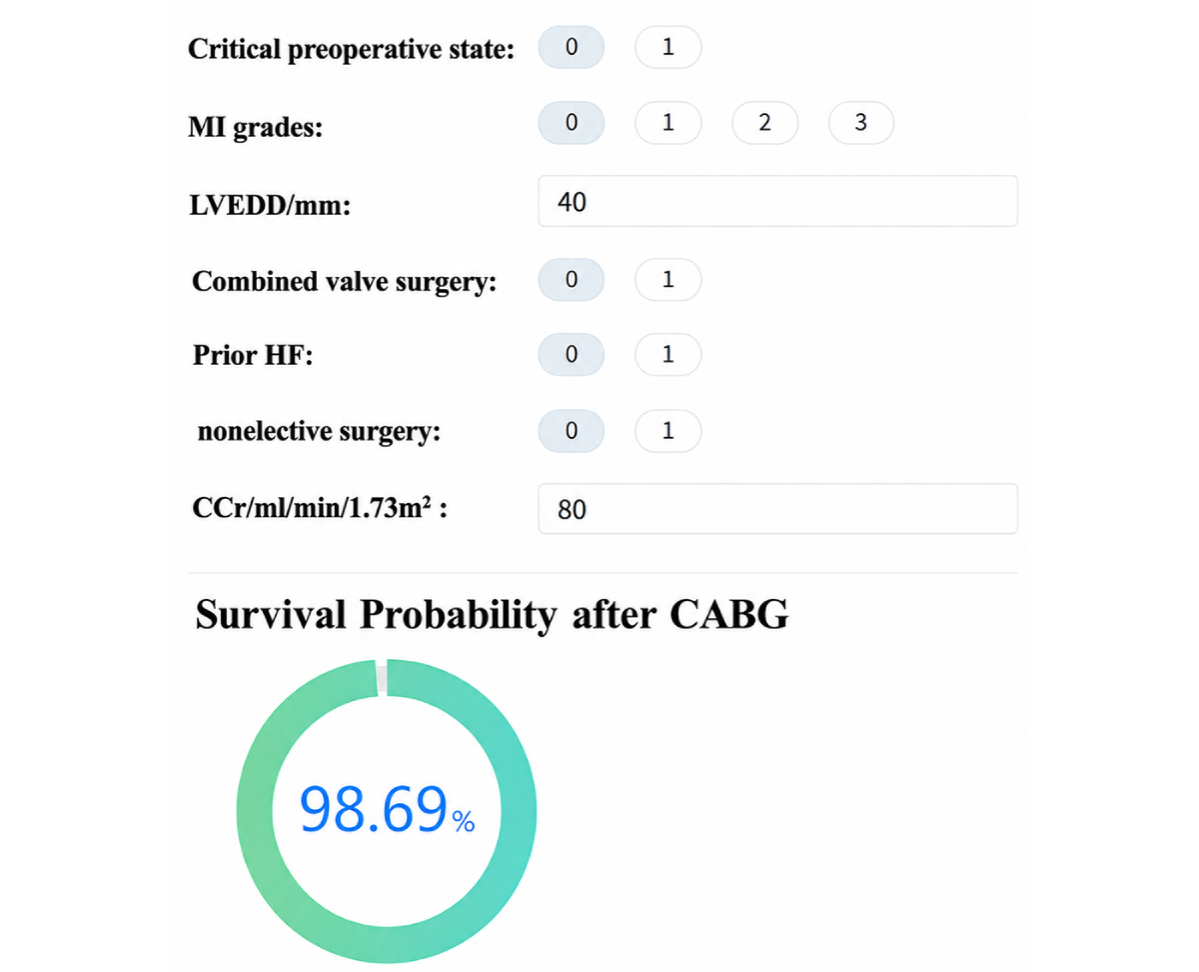
Example of using the web calculator. LVEDD: left ventricular end-diastolic diameter.

Based on the optimal XGBoost model, a web-based risk calculator was developed to facilitate clinical use (Figure 8). By entering the values of the 7 key variables, the tool calculates the specific probability of in-hospital mortality. For the representative patient shown in Figure 8, the calculator estimates a mortality risk of 1.31% (corresponding to a 98.69% survival probability). This numerical output may offer auxiliary information for clinicians during risk assessment, providing a data-driven reference alongside traditional scoring systems.

**Table 2:** Performance of machine learning models in the training cohort.

Model	AUC^a^	*F*_1_-score	Recall	Precision	Sensitivity	Specificity	Accuracy
Adaptive boosting	0.816	0.000	0.000	0.000	0.000	1.000	0.977
Bernoulli Naive Bayes	0.777	0.136	0.082	0.402	0.082	0.997	0.976
Gaussian Naive Bayes	0.778	0.145	0.383	0.089	0.383	0.909	0.897
DT^b^	0.783	0.108	0.610	0.059	0.610	0.773	0.769
GB^c^	0.831	0.066	0.035	0.824	0.035	1.000	0.978
KNN^d^	0.991	0.181	0.104	0.700	0.104	0.999	0.979
LSVC^e^	0.800	0.112	0.686	0.061	0.686	0.753	0.751
LDA^f^	0.793	0.197	0.180	0.217	0.180	0.985	0.977
LR^g^	0.800	0.110	0.696	0.059	0.696	0.743	0.742
RF^h^	1.000	0.988	0.995	0.981	0.995	1.000	0.999
SGD^i^	0.617	0.000	0.000	0.000	0.000	1.000	0.977
XGBoost^j^	0.850	0.085	0.044	0.857	0.044	1.000	0.978
MLP^k^	0.814	0.057	0.030	0.800	0.030	1.000	0.978
SVM^l^	0.819	0.000	0.000	0.000	0.000	1.000	0.977

^a^AUC: area under the receiver operating characteristic curve.

^b^DT: decision tree.

^c^GB: gradient boosting.

^d^KNN: k-nearest neighbor.

^e^LSVC: linear support vector classification.

^f^LDA: linear discriminant analysis.

^g^LR: logistic regression.

^h^RF: random forest.

^i^SGD: stochastic gradient descent.

^j^XGBoost: Extreme Gradient Boosting.

^k^MLP: multilayer perceptron.

^l^SVM: support vector machine.

**Table 3:** Performance of the machine learning models in the validation cohort.

Model	AUC^a^	*F*_1_-score	Recall	Precision	Sensitivity	Specificity	Accuracy
Adaptive boosting	0.793	0.000	0.000	0.000	0.000	1.000	0.977
Bernoulli Naive Bayes	0.765	0.138	0.084	0.391	0.084	0.997	0.976
Gaussian Naive Bayes	0.775	0.146	0.383	0.090	0.383	0.910	0.898
DT^b^	0.729	0.095	0.583	0.052	0.583	0.750	0.746
GB^c^	0.798	0.047	0.025	0.417	0.025	0.999	0.977
KNN^d^	0.611	0.059	0.032	0.342	0.032	0.999	0.977
LSVC^e^	0.795	0.110	0.677	0.060	0.677	0.752	0.750
LDA^f^	0.787	0.195	0.178	0.216	0.178	0.985	0.967
LR^g^	0.623	0.068	0.047	0.123	0.047	0.992	0.971
RF^h^	0.710	0.000	0.000	0.000	0.000	1.000	0.910
SGD^i^	0.559	0.084	0.049	0.270	0.049	0.997	0.975
XGBoost^j^	0.798	0.046	0.025	0.385	0.025	0.999	0.977
MLP^k^	0.796	0.070	0.037	0.682	0.037	1.000	0.978
SVM^l^	0.761	0.000	0.000	0.000	0.000	1.000	0.977

^a^AUC: area under the receiver operating characteristic curve.

^b^DT: decision tree.

^c^GB: gradient boosting.

^d^KNN: k-nearest neighbor.

^e^LSVC: linear support vector classification.

^f^LDA: linear discriminant analysis.

^g^LR: logistic regression.

^h^RF: random forest.

^i^SGD: stochastic gradient descent.

^j^XGBoost: Extreme Gradient Boosting.

^k^MLP: multilayer perceptron.

^l^SVM: support vector machine.

## Discussion

### Principal Findings

Cardiovascular disease is a major public health issue, of which ischemic heart disease is one of the most prevalent conditions, with a high prevalence and a substantial proportion of patients requiring surgical treatment. CABG is the primary surgical treatment for ischemic heart disease [4]. According to the STS ACSD, advances in surgical techniques and multidisciplinary management have kept the operative mortality for CABG consistently at a low level, ranging from 1.76% to 2.08% between 2015 and 2022 [5]. Establishing a postoperative mortality risk assessment system for CABG can significantly benefit surgeons by enabling them to accurately identify high-risk cases and target perioperative management to reduce risks. Additionally, such a system can assist health administrations in understanding overall diagnostic and therapeutic levels, guiding the formulation and implementation of relevant policies.

Patients included in this study were selected from the CCSR database, a nationwide multicenter registry established in 2013. The CCSR aims to enable continuous quality monitoring of adult cardiovascular surgery, drive quality improvement efforts, and foster a collaborative network for clinical research at the national level. The CCSR database includes data from consecutive patients undergoing cardiac surgery at 87 participating centers and captures approximately 30% to 40% of all CABG procedures and valve operations and reflects performance in large cardiac centers [8]. The in-hospital mortality rate for isolated CABG in our study was 1.5%. It is close to the 1.6% mortality rate reported by Yuan et al [19] for isolated CABG in urban teaching hospitals in China between 2004 and 2013. It is also slightly lower than the mortality rate reported for isolated CABG in the STS ACSD [5]. The overall mortality rate was 2.1% (450/21,443), partly because several included patients underwent combined cardiovascular surgery, such as valve surgery, which resulted in more complex cardiac conditions, poorer cardiac function, and higher mortality risk [9,20]. Perioperative management for patients undergoing combined surgery requires more clinical attention. The prediction model developed in this study can more accurately predict the mortality risk in this group of patients, offering more precise assessment and treatment options. Our methodology prioritized the stability of variables through a preliminary ICC-based filter. Unlike simple univariate screening, this approach ensures that the model inputs are consistent across different data slices, reducing the risk of overfitting to random fluctuations within the dataset before applying the LASSO selection.

Risk factors for operative mortality after CABG have been a critical concern for cardiac surgeons. Identifying and controlling these key risk factors can enhance surgical practices, improve patient survival, and lead to better overall patient prognosis. The risk factors incorporated in the STS risk models, EuroSCORE Ⅱ, and the SinoSCORE include age, NYHA classification, renal function, LVEF, critical preoperative status, emergency surgery, gender, COPD, and diabetes mellitus [9-11]. The risk model for in-hospital mortality after CABG, established by Hu et al [21], added additional variables such as prior cardiac surgery, prior PCI, prior cerebrovascular accident, and combined surgery to these prediction factors. Variables included in our XGBoost model were prior HF, critical preoperative state, CCr, LVEDD, MI, combined valve surgery, and nonelective surgery. Notably, traditional risk factors such as age and LVEF, which are central to the EuroSCORE II and STS models, were not selected in our final parsimonious model. This is likely because the nonlinear XGBoost algorithm captured their predictive value through other correlated variables. For instance, age is mathematically incorporated into the CCr calculation (Cockcroft-Gault formula), which the model identified as a stronger predictor of physiological reserve. Similarly, LVEDD was selected over LVEF. Previous studies have suggested that left ventricular dilation (LVEDD) may serve as a more sensitive marker of chronic remodeling and long-term prognosis than ejection fraction in ischemic cardiomyopathy [22,23]. When comparing performance, our model (test AUC 0.782) outperformed EuroSCORE II (test AUC 0.722) and SinoSCORE (test AUC 0.726). We acknowledge that EuroSCORE II predicts 30-day mortality while our outcome is in-hospital mortality. Although this end point mismatch limits a direct “apples-to-apples” comparison, the lower discrimination of EuroSCORE II in this Chinese cohort also suggests that locally calibrated models derived from machine learning may offer superior precision for this specific population. Jie et al [22] conducted a follow-up study on patients with three-vessel disease who underwent CABG and found that those with significant left ventricular dilatation (LVEDD >53 mm) had a significantly higher long-term mortality (15.4% vs 4.8%). Yamaguchi et al [23] similarly found that left ventricular dilatation was an independent risk factor for congestive heart failure after CABG. Left ventricular dilation indicates weakened cardiac systolic function and may be associated with mitral regurgitation, which can result from primary or secondary factors, including ischemia. Some patients may require combined valve surgery, which carries a higher risk of postoperative heart failure and increases the likelihood of operative mortality following CABG.

Currently, several risk prediction models for postoperative mortality after CABG are used, including the STS risk model, EuroSCORE II, and SinoSCORE [9-11]. These traditional models typically use classical multifactorial analysis methods, such as logistic regression. While these models are broadly applicable, they have inherent limitations, including restricted inclusion of risk factors, limited potential for improvement, and lack of transparency [24-26]. Most existing models rely on clinical data from 10 to 20 years ago, which may not accurately reflect the current state of medical practice of ischemic heart disease or the differences in morbidity characteristics between Eastern and Western populations. The XGBoost prediction model developed in this study demonstrated superior performance, achieving AUCs of 0.850 (95% CI 0.832-0.868) in the training cohort and 0.782 (95% CI 0.709-0.855) in the test cohort. This performance exceeds that of the EuroSCORE Ⅱ and SinoSCORE and offers a more accurate assessment of operative mortality risk for CABG in the Chinese population. As the sample size expands, the advantage of machine learning is likely to become more evident, and its applicability and stability could be further explored across different regions and populations.

AI is a branch of computer science focused on replicating human thought processes, learning abilities, and knowledge storage [27]. As a rapidly evolving technology, AI has been increasingly applied in the medical field. Machine learning is the core technology and implementation method of AI. It involves developing computer algorithms to analyze data, deduce, summarize, and discover underlying patterns. Through self-learning, training, and iterative modification, machine learning algorithms continuously enhance their accuracy [14,28]. Developing predictive models for disease risk and prognosis is a key application of machine learning in clinical research. Machine learning excels in handling large samples, multimodal data, and personalized assessments. Moreover, ongoing advancements in machine learning algorithms continue to expand their applicability to a wider range of scenarios. Several studies have applied machine learning to cardiac surgery related risk prediction. These applications include predicting postoperative mortality risk and the likelihood of complications, such as acute kidney injury, AF, and readmission [29-32]. In recent years, research on the application of machine learning has become increasingly systematic. While machine learning models offer advantages, such as overcoming limitations of traditional models, high flexibility, and broad applicability, there may be new challenges, such as overfitting, poor interpretability, extended training times, and higher computational requirements [33]. In our study, the near-perfect training AUCs for RF and k-nearest neighbor reflect the inherent complexity of these models on imbalanced data, serving as a cautionary benchmark for overfitting. By prioritizing the performance in the independent test cohort, we identified XGBoost as the most generalizable algorithm, emphasizing that test performance is the only reliable metric for potential clinical translation. This approach underscores the necessity of using independent datasets to filter out overfitted models before considering real-world application. Frizzell et al [30] applied various machine learning algorithms, such as RF and gradient boosting, to develop a model for predicting the risk of 30-day readmission in patients hospitalized with HF. They ultimately found that the machine learning model did not demonstrate better predictive performance compared with traditional prediction models. Our research team has extensive experience in developing predictive models, applying traditional logistic regression and various machine learning algorithms to create multiple models for assessing the risk of mortality and complications associated with cardiac surgery. We used the above methods to develop a prediction model for the risk of in-hospital mortality after valve surgery in older patients. Our findings showed that both approaches had satisfying predictive performance, with no statistically significant differences between them [34]. The study suggests that both traditional logistic regression and advanced machine learning algorithms have unique advantages and disadvantages in prediction model development. These methods are suitable for different sample sizes, data types, and application scenarios. It is essential to use multiple methods to build the model, based on comprehensive data collection, collation, and summarization. Ultimately, the model with the best predictive performance should be selected by comparing discrimination and calibration indicators.

Our study has several limitations. First, the primary end point was in-hospital mortality due to the current data structure of the CCSR, rather than the 30-day mortality used in EuroSCORE II. Crucially, while our test cohort was independent of the training process, it was derived from the same registry. Therefore, our results represent internal-independent validation rather than true external validation. The lack of geographic or temporal separation between the training and test sets means that the model’s performance in different health care environments remains to be proven. Second, while the CCSR covers 87 centers, these are predominantly large academic centers, potentially introducing selection bias regarding rural or nonteaching hospitals. Third, despite addressing class imbalance with SMOTE, the relatively low number of mortality events in the test cohort results in wider CIs for our performance metrics. Fourth, the “perfect” receiver operating characteristic curves observed for RF and k-nearest neighbor in the training phase indicate a tendency toward overfitting in complex models, although the chosen XGBoost model demonstrated stable performance in the independent test cohort. Future studies will aim to incorporate long-term follow-up and imaging biomarkers to further refine the model.

### Conclusions

Our study developed and validated a machine learning–based risk prediction model for in-hospital mortality after CABG using a large-scale Chinese multicenter registry. Among the algorithms tested, the XGBoost model demonstrated superior discrimination and calibration compared with the traditional EuroSCORE II and the SinoSCORE, suggesting that locally calibrated models may better capture the risk profile of Chinese patients. The 7-variable web calculator is presented as a pilot tool for clinician reference. Future research must prioritize true external validation across diverse hospital tiers and geographic regions to establish the model’s reliability before clinical implementation.

## Abbreviations

ACSD: Adult Cardiac Surgery Database
AF: atrial fibrillation
AI: artificial intelligence
AUC: area under the receiver operating characteristic curve
CABG: coronary artery bypass grafting
CCr: creatinine clearance rate
CCS: Canadian Cardiovascular Society
CCSR: Chinese Cardiac Surgery Registry
CKD: chronic kidney disease
COPD: chronic obstructive pulmonary disease
EuroSCORE II: European System for Cardiac Operative Risk Evaluation II
HF: heart failure
ICC: intraclass correlation coefficient
LASSO: Least Absolute Shrinkage and Selection Operator
LVEDD: left ventricular end-diastolic diameter
LVEF: left ventricular ejection fraction
MI: myocardial infarction
NYHA: New York Heart Association
PCI: percutaneous coronary intervention
PVD: peripheral vascular disease
RF: random forest
SinoSCORE: Sino System for Coronary Operative Risk Evaluation
SMOTE: synthetic minority oversampling technique
STS: Society of Thoracic Surgeons
XGBoost: Extreme Gradient Boosting

## Data Availability

The data presented in this study are available upon request from the corresponding author. The data are not publicly available due to the No. 717 Chinese Decree of the State Council, which states that the raw data from large samples cannot be shared globally.
